# Orbital myiasis on recurrent undifferentiated carcinoma in the COVID-19 era: a case report and brief review of the literature

**DOI:** 10.1186/s12348-021-00271-1

**Published:** 2021-11-20

**Authors:** Mansooreh Jamshidian-Tehrani, Kasra Cheraqpour, Mohammad Amini, Fahimeh Asadi Amoli, Abolfazl Kasaee

**Affiliations:** Eye Research Center, Farabi Eye Hospital, Tehran University of Medical Sciences, Qazvin Square, Tehran, 1336616351 Iran

**Keywords:** Myiasis, Cancer, COVID-19, Orbital myiasis, Undifferentiated carcinoma

## Abstract

**Background:**

Myiasis is defined as the infestation of living tissues by Diptera larvae. Ophthalmic involvement occurs in less than 5% of cases. As the most uncommon type of involvement, orbital myiasis usually affects patients with poor personal hygiene, a low socioeconomic status, a history of surgery, and cancer.

**Findings:**

In January 2020, an 89-year-old man presented to the Oculoplastic Department of Farabi Eye Hospital (Iran) with a history of left-side progressive orbital mass for six months. A large infiltrative mass of the left orbit with extension to the globe, periorbita, and adnexa was remarkable at the presentation, and its appearance suggested malignancy. Our findings persuaded us to perform exenteration and histopathological evaluation which were reported as “undifferentiated carcinoma”. Regular follow-up visits were recommended. In June 2020, with a 3-month delay, the patient presented with the recurrence of the mass complicated with mobile alive larva. Examinations revealed numerous maggots crawling out of an ulcerative and foul-smelling lesion. He stated that fear of COVID-19 infection postponed his follow-up visit. The patient underwent immediate mechanical removal of larvae, followed by wide local excision of the mass.

**Conclusion:**

Patients with carcinoma of the adnexal tissues seem to be more prone to myiasis infestation even though it is an uncommon disease. Since COVID-19 is an ongoing pandemic with no end in sight appropriate protocols should be implemented to prevent loss of follow-up in these high risk patients.

**Supplementary Information:**

The online version contains supplementary material available at 10.1186/s12348-021-00271-1.

## Introduction

‘Myiasis’ is derived from ‘myia’, a Greek word meaning fly [1]. This condition involves the infestation of living tissues by Diptera larvae. Ophthalmomyiasis which is responsible for less than 5% of cases can be occasionally a life-threatening condition. Crowded places, poor personal hygiene, a low socioeconomic status, surgery, cancer, ischemia, and infection can make patients prone to orbital myiasis [2]. Myiasis is more prevalent in rural regions with a warm climate. Animals such as goats and sheep are natural hosts, whereas humans are invaded accidentally. Myiasis has a destructive behavior, which necessitates early diagnosis and immediate intervention [3].

Limited reports are available in the literature on orbital myiasis. Herein, we report a rare case of orbital myiasis in an old man with a history of orbital tumor that missed his follow-up visits due to the COVID-19 pandemic. Eventually, he presented with the recurrence of the tumor complicated with orbital myiasis.

## Case presentation

In January 2020, an 89-year-old man presented to the Oculoplastic Department of Farabi Eye Hospital with a history of left-side progressive orbital mass for six months. At the presentation, the best-corrected visual acuity (BCVA) of the right and the left eyes was 20/100 and no light perception (NLP), respectively. Examinations of the right eye revealed dermatochalasis and 3+ nuclear sclerosis cataract. Other slit and fundus examinations of the right eye were normal. The examination of the left globe was not possible due to a large infiltrative mass with extension to the globe, periorbita, and adnexa. The patient’s history was negative for systemic disease or malignancy. The appearance of the mass was suggestive of malignancy more than other diagnoses. Our findings persuaded us to perform exenteration and histopathological evaluations which were reported as “undifferentiated carcinoma in some foci admixed with sarcomatous stroma (carcinosarcoma), most probably metastatic in origin”. An immunohistochemistry (IHC) study showed weakly positive EMA, positive Pan CK, CK-HM, and CK-LM, diffusely positive vimentin, positive CK7, and negative CK20 and S100 in tumoral cells. An oncology consultation was requested to investigate the possible origin of the tumor. To date, all of the investigations were negative.

**Fig. 1:**
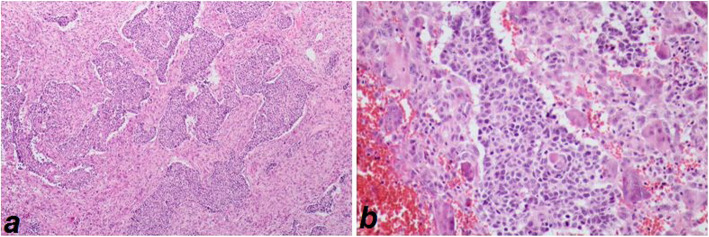
Islands of undifferentiated carcinoma in the stroma with diffuse foreign body type granulomatous response, H&E stained (×100,400)

The patient was asked to visit in March 2020, a date that coincided with the beginning of the COVID-19 epidemic in Iran, leading to quarantine policies and measures. This condition coupled with his poor compliance delayed his follow-up visit. In June 2020, the patient presented after a 3-month delay with recurrence of his mass complicated by numerous maggots crawling out of an ulcerative and foul-smelling lesion. He stated that fear of the COVID-19 pandemic had caused him to postpone his follow-up visits. The size of the lesion was approximately 10 × 8 cm. We used a single dose of oral ivermectin prior to surgery to reduce the difficulty of the mechanical removal of the maggots. After oncology consultation, a wide local excision was planned. The specimen for the histopathological study showed interesting findings: undifferentiated carcinoma in some foci in the stroma, with diffuse foreign body-type granulomatous inflammation containing many multinucleated giant cells (Fig. 1). During the surgery, more than 50 maggots were removed from the mass surface (Video 1). In the postoperative period, the patient underwent close observation for monitoring wound infection and removing the missed larvae. Once the wound began to heal, he was discharged.

## Discussion

FW Hope described myiasis for the first time as the infestation of living tissues of vertebrate animals by fly larvae of Diptera called maggots [4, 5]. Presentations may differ from absence of symptoms to death. The number of invading larvae depends on the genus of Diptera and may vary from one to more than a hundred [6]. Ophthalmic involvement, known as ophthalmomyiasis, occurs in less than 5% of cases. Ophthalmomyiasis can be classified into external (limited to palpebral and conjunctival tissues), internal (penetration of conjunctiva and sclera with subretinal tissue involvement), and orbital which is a less common but the most severe type [7]. Ophthalmomyiasis usually affects patients on both sides of the age distribution. Poor socioeconomic status, overcrowding, poor sanitation and personal hygiene, alcoholism, debilitating conditions, and mental illness may make the individuals prone to myiasis [8]. The predisposing factors of our case were rural background and low socioeconomic status. We also implicate the COVID-19 pandemic and its psychological effects as stated by the patient. The relatively high prevalence of myiasis in Iran may be justified by the subtropical condition of some provinces and the remarkable number of rural residents in contact with domestic animals. Orbital myiasis usually manifests as unilateral involvement, with itching and crawling sensations are the most common symptoms [9].

Up to 80 species of Diptera can infest human tissues. *Dermatobia hominis* (human botfly), *Cochliomyia hominivorax* (screw worm), *Hypoderma bovis* (ox warble fly), and *Oestrus ovis* (sheep botfly) are accounted as the most common species [10]. The larvae may invade several parts of the eye [7]. The Dipteral larvae are photosensitive, and this is the underlying reason for their deep penetration into the tissues [3]. An interesting hypothesis is that adult larvae may select their hosts through visual or olfactory stimuli by attraction to fresh blood. This is usually observed in skin malignancies. Exposed suppurative, necrotic, or hemorrhaging lesions are attractive factors for flies. Hence, the debridement of cellular debris and nonviable necrotic tissue from the wound bed and keeping the wound clean by regular bandage changing can reduce the chance of infection. Previously, skin cancers were implicated as a predisposing factor for orbital myiasis [7]. Among the reported cases of malignancies complicated by orbital myiasis, basal cell carcinoma seems to be more prevalent [2]. Table 1 summarizes similar reports of orbital myiasis on cancers.

**Table 1:** Previous similar reports of orbital myiasis in ophthalmic cancers

No. [Ref]	Age/Sex	Predisposing factors	Eye	Underlying cancer	Management	Species/Number
1[7]	85/F	*Malignancy	OD	Recurrent basal cell carcinoma of the inferior eyelid	Exenteration plus maxillectomy	*Hypoderma bovis*/ 71
2[2]	73/M	*Rural background *Significant bed of necrosis *Poor general condition *Lack of self-care *Overcrowded environment	OS	Basal cell carcinoma of the lower eyelid with invasion into the orbit	Manual removal and debridement followed by antibiotics an ivermectin	Not mentioned/ 100
3[11]	98/M	*Significant bed of necrosis *Poor general condition *Several medical comorbidities *Old ages *Poor hygiene	OD	Squamous cell carcinoma of the eyelids	Despite aggressive treatment, the patient died within 8 h.	Sarcophaga argyrostoma/ Not mentioned
4[12]	85/M	*Mental disease *Dehydration *Cachexia *Poor hygiene	OS	Basal cell carcinoma	Manual removal followed by antibiotics and exenteration. (After surgery, the patient passed away due to cardiopulmonary failure)	Lucilia sericata/ About 70
5[1]	74/F	*Malignancy	OS	Basal cell carcinoma	Antibiotics and ivermectin followed by debridement and enucleation.	*Cochliomyia hominivorax*/ About 100
6[13]	24/M	*Significant bed of necrosis *Poor hygiene *Low socioeconomic status *Defective immunity due to xeroderma pigmentosa	OS	Squamous cell carcinoma	Exenteration	Calliphoridae/ Not mentioned
7[14]	83/M	*Malignancy	OS	Basal cell carcinoma	Manual removal followed by antibiotics	Not mentioned/ Not mentioned
8[15]	80/M	*Malignancy	OD	Squamous cell carcinoma of the eyelid	Manual removal followed by antibiotics	*Cochliomyia hominivorax*/ Not mentioned
9[8]	50/M	*Malignancy	OS	Basal cell carcinoma	Manual removal followed by exenteration	Phylum Arthropoda/ Not mentioned
10[16]	2/ Not mentioned	*Significant bed of necrosis *Lack of self care and communication	OD	Retinoblastoma	Manual removal followed by antibiotics, radiotherapy and chemotherapy	Not mentioned/ Not mentioned

The diagnosis is based on clinical findings, and the treatment aims to remove all of the larvae and prevent bacterial superinfection [7]. Brain, orbital, and paranasal sinuses’ CT scan/MRI is usually requested to determine the extension of involvement [2]. Solutions such as turpentine oil, liquid paraffin, and petroleum jelly can immobilize the larvae and facilitate the mechanical removal of maggots [3]. The other facilitating options include hydrogen peroxide and isopropyl alcohol with larvicidal properties [3]. Recently, ivermectin has been used for treatment, and its use prior to the surgery has been recommended to facilitate the mechanical removal of the maggots. In fact, much of the experience of the use of ivermectin for myiasis treatment comes from veterinary medicine. In other words, ivermectin is an off-label option in many countries since no standard study has been conducted on its use. Although a single oral dose of ivermectin is the most commonly prescribed form of usage, the role of continuing this agent after surgical debridement to reduce the chance of recurrence is still not clear. As for surgical management, options vary from saving the globe approach through surgical removal of larvae to aggressive surgeries such as exenteration [2]. Note that the mechanical removal of the flies may be required in more than one session [3]. The chance of intracranial invasion due to the proximity of the globe to the brain highlights the significance of orbital myiasis, differentiating it from the infestation of other sites. Therefore, exenteration is recommended in cases with orbital apex involvement.

## Conclusion

Patients with carcinoma of the adnexal tissues seem to be more prone to myiasis infestation even though it is an uncommon disease. Since COVID-19 is an ongoing pandemic with no end in sight appropriate protocols should be implemented to prevent loss of follow-up in these high risk patients.

## Supplementary Information

Additional file 1: Video 1. Showing numerous maggots crawling out of an ulcerative lesion with necrotic surface.

## Data Availability

The data of current case report are available from the corresponding author on reasonable request.

## Abbreviations

BCVA: Best-corrected visual acuity
NLP: No light perception
IHC: Immunohistochemistry
CT scan: Computed tomography scan
MRI: Magnetic resonance imaging
M: Male
F: Female
OD: Oculus dexter (right eye)
OS: Oculus sinister (left eye)
